# Long term prevention of disturbance induces the collapse of a dominant species without altering ecosystem function

**DOI:** 10.1038/srep14320

**Published:** 2015-09-21

**Authors:** Qiang Yu, Honghui Wu, Zhengwen Wang, Dan F. B. Flynn, Hao Yang, Fumei Lü, Melinda Smith, Xingguo Han

**Affiliations:** 1 State Key Laboratory of Forest and Soil Ecology, Institute of Applied Ecology, Chinese Academy of Sciences, Shenyang 110016, China; 2 Department of Biology, Graduate Degree Program in Ecology, Colorado State University, Fort Collins, Colorado 80523 USA; 3 Global Change and Alpine Ecosystem Research Laboratory, Northwest Institute of Plateau Biology, Chinese Academy of Sciences, Xining 810008, China; 4 Institute of Evolutionary Biology and Environmental Studies, University of Zurich, 8057 Zurich, Switzerland; 5 Institute of Geographic Sciences and Natural Resources Research, Chinese Academy of Sciences, Beijing 100101, China; 6 Shenzhen Baoan Qianlong School, Shenzhen 518131, China; 7 State Key Laboratory of Vegetation and Environmental Change, Institute of Botany, Chinese Academy of Sciences, Beijing 100093, China

## Abstract

Limitation of disturbances, such as grazing and fire, is a key tool for nature reserve management and ecological restoration. While the role of these disturbances in shaping ecosystem structure and functioning has been intensively studied, less is known about the consequences of long-term prevention of grazing and fire. Based on a 31-year study, we show that relative biomass of the dominant grass, *Leymus chinensis,* of grasslands in northern China declined dramatically, but only after 21 years of exclusion of fire and grazing. However, aboveground net primary productivity (ANPP) did not decline accordingly due to compensatory responses of several subdominant grass species. The decline in dominance of *L. chinensis* was not related to gradually changing climate during the same period, whereas experimentally imposed litter removal (simulating fire), mowing (simulating grazing), fire and moderate grazing enhanced dominance of *L. chinensis* significantly. Thus, our findings show that disturbances can be critical to maintain the dominance of key grass species in semiarid grassland, but that the collapse of a dominant species does not necessarily result in significant change in ANPP if there are species in the community capable of compensating for loss of a dominant.

Ecosystems are being degraded and biodiversity is declining because of global changes, such as land use change, altered biogeochemical cycles and disturbance regimes, and climate change, resulting from human activities^1^,^2^. Considerable research has shown that disturbances (e.g., mowing, fire and grazing) impact most ecosystems to some degree and in some cases are even required for ecosystem persistence^3^,^4^,^5^,^6^,^7^. However, conservation and restoration efforts often employ means to prevent disturbances, both natural and human-caused^8^,^9^, to counter trends of ecosystem degradation and biodiversity loss. While severely degraded ecosystems can recover rapidly after disturbances are removed^8^, biodiversity and ecosystem services decreased with prevention of disturbances in undegraded ecosystems^10^,^11^,^12^, suggesting that long-term protection of degraded ecosystems might have negative effects^4^. Two long-term studies showed that prevention of disturbances changed ecosystem structure^13^ and the accumulated litter decreased species richness and evenness^14^. However, our understanding is limited concerning the long-term (e.g., >30 years) impacts of removal of disturbances on plant community dynamics and ecosystem function for ecosystems in which disturbances are integral to their structure and function. Long term studies on dynamics of these “protected” ecosystems are needed, especially within the backdrop of global change.

Disturbances generally impact ecosystems by altering biotic structure^15^, and it is the changes in community structure and associated biogeochemical processes that drive subsequent ecosystem dynamics over the long-term^16^. In some cases, ecosystem recovery from disturbance can be rapid^8^, whereas in other cases it can be prolonged, particularly if species that contribute disproportionately to ecosystem function (i.e., dominant species^17^) are negatively impacted 18,19,20,21. Grazing and fire are disturbances in grassland that are often modified by humans, with these alterations ranging from increased frequency to complete exclusion^22^. The long-term prevention of grazing and fire can have varying impacts as well, depending on the degree to which the disturbance(s) being excluded impacts dominant species^4^. The expectation is that if the exclusion of grazing and fire does not favor dominant species and/or strongly modifies biogeochemical processes, then the ecosystem impacts of the disturbance prevention can be large and potentially nonlinear, particularly if disturbance is excluded over the long-term and in tandem with other global change drivers, such as climate change^22^. Thus, it remains unclear whether ecosystem structure and function changes gradually (linearly) or abruptly (nonlinearly) after long-term prevention of disturbance and whether shifts in abundance of dominant species drive these dynamics.

Here, we examine the impacts of long-term exclusion of two key disturbance regimes – fire and grazing–in a semi-arid grassland in Inner Mongolia. *Leymus Chinensis*, a C_3_ rhizomatous grass, is the dominant plant species in these grasslands. Historically, Inner Mongolia grasslands experienced an intermediate disturbance frequency^23^, but these grasslands have more recently been degraded because of heavy grazing and alterations in fire regimes (primarily fire exclusion). Our study addresses the following questions: (1) Whether dominance of *L. chinensis*, plant community structure, and ecosystem functioning (aboveground net primary productivity, ANPP) changes gradually or abruptly during long-term (30 + years) exclusion of two key disturbances – fire and grazing? and (2) Does the shift of dominant species result in significant change in ecosystem function? Our expectation was that long-term exclusion of disturbances would negatively impact dominance by *L. chinensis* over time, and that this decline in dominance would alter plant community structure and negatively impact ANPP. The goal of our research was to document these dynamics and assess potential mechanisms underlying the expected decline in *L. chinensis* dominance.

## Results

**Figure 1:**
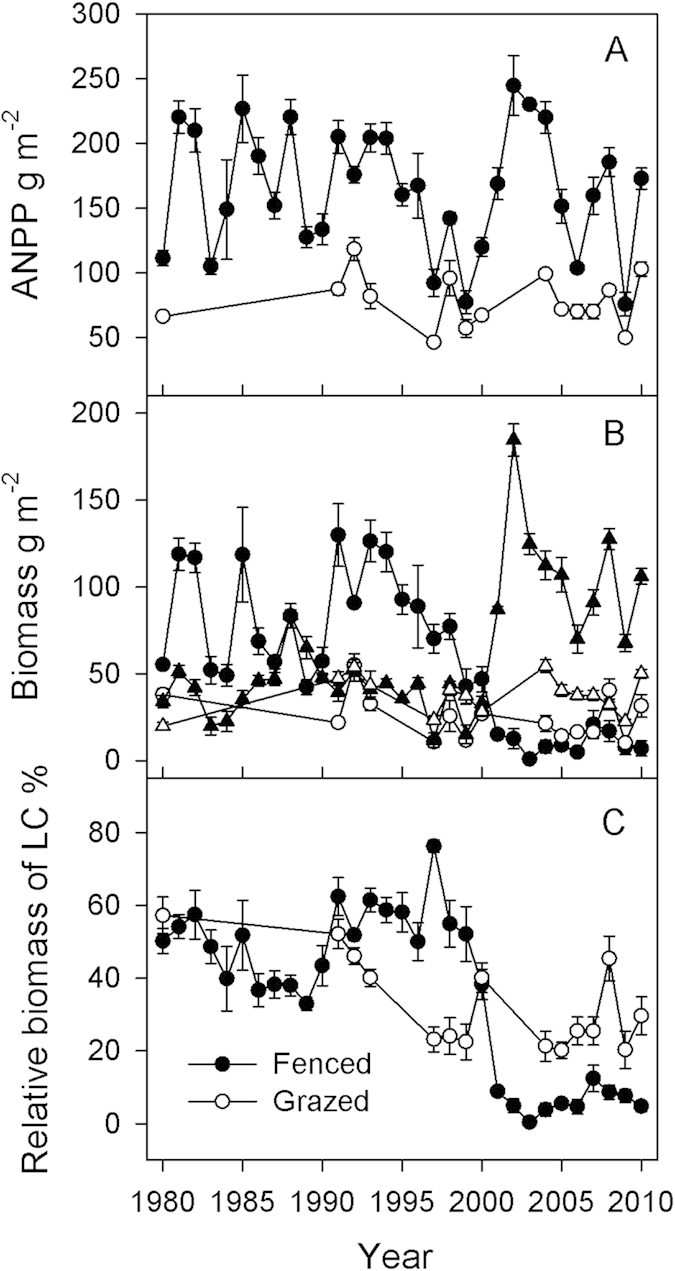
Dynamics of biomass of LC and perennial bunchgrasses (PB, A), aboveground net primary productivity (ANPP, B), and relative biomass of *Leymus chinensis* (LC, C), during the past 31 years in an Inner Mongolian grassland. Data in fenced site are showed with filled symbols, and data in grazed site are showed with open symbols. Dots represent LC, and triangles represent PB in (**B**).

The long-term (31 years) exclusion of fire and grazing disturbances resulted in the degree of dominance of *L. chinensis* varying in two distinct periods: 1) dominant from 1980–2000 and 2) then sub-dominant from 2001–2010 (Fig. 1A,C). In the period from 1980–2000, biomass of *L. chinensis* changed within a range of 42.27–129.84 g m^−2^. However, it declined sharply from 1997, reaching a minimum in 2003, and then fluctuated between 0.88–20.86 g m^−2^ during 2001–2010 (Fig. 1A). In contrast, biomass of perennial bunchgrasses (PB) increased from a range of 11.74–83.82 g m^−2^ during 1980–2000 to 70.00–184.57 g m^−2^ during 2001–2010 (Fig. 1A). However, biomass of *L. chinensis* and PB in the grazed area remained relatively stable compare to fenced area. Despite the large change in biomass of *L. chinensis* in fenced area, aboveground net primary productivity (ANPP) did not exhibit a significant decline over the study period, although was variable over time (Fig. 1B). ANPP in the fenced area decreased with the decrease of *L. chinensis* from 1997–2000, while increased with the increase of PB from 2000–2003. ANPP in the grazed area was much lower than that in fenced area. In the period from 1980–2000, the relative abundance (biomass) of *L. chinensis* in the fenced area was on average 50.25 ± 2.32 (Mean ± SE, Fig. 1C), varying from 33% to 76%. However, it declined sharply from 1997, reaching a minimum in 2003, and then fluctuated between 0.4%–12.4% (6.18 ± 0.75). The relative biomass of *L. chinensis* in the grazed area was lower than that in fenced area from 1980–2000, however, higher from 2001–2010, with no clear trend of increase or decrease.

**Figure 2:**
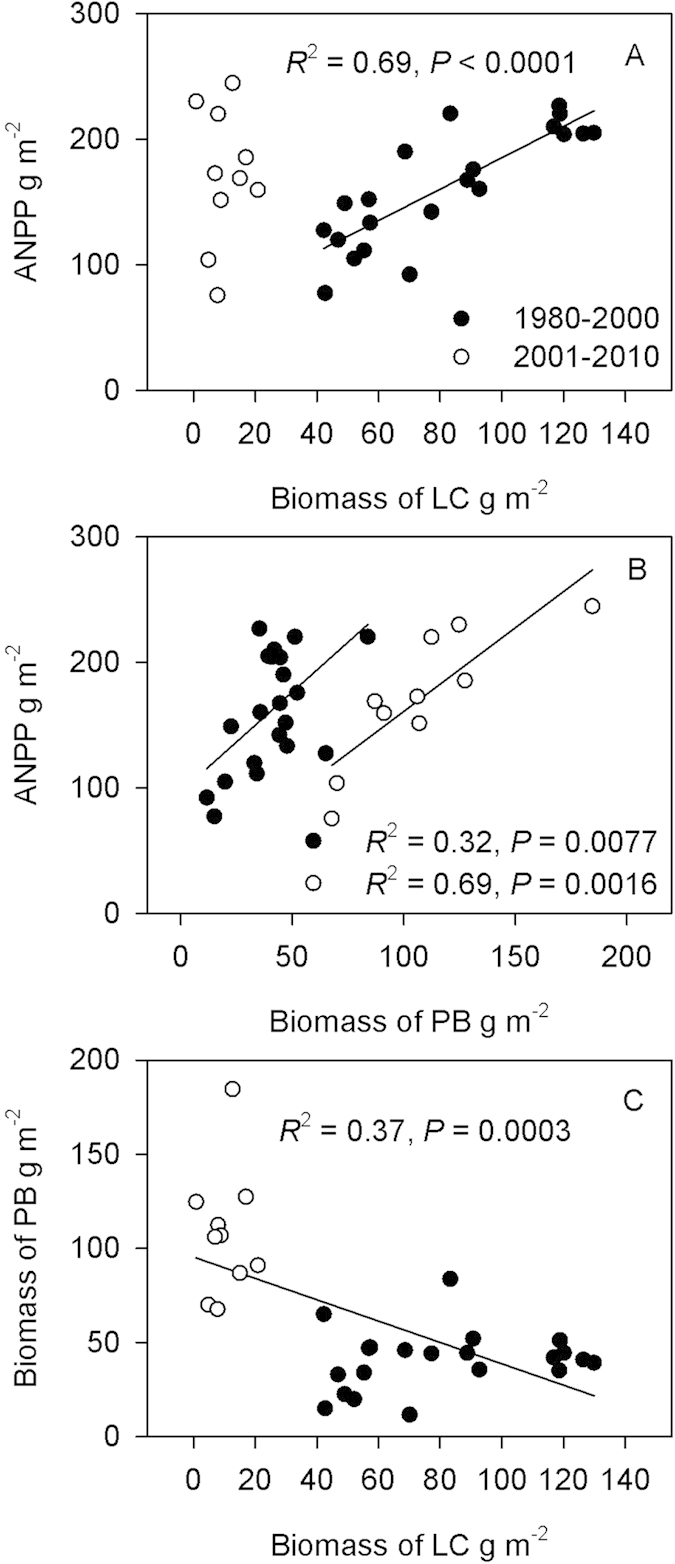
Relationships between biomass of *Leymus chinensis*, biomass of perennial bunchgrasses (PB), and aboveground net primary productivity (ANPP).

Biomass of *L. chinensis* contributed more than 50% of ANPP in the fenced area from 1980–2000 (Fig. 1A), and explained 69% variation in ANPP during this period (Fig. 2A). However, there was no significant relationship between *L. chinensis* biomass and ANPP during 2001–2010 (Fig. 2A). Biomass of PB was significantly and positively related with ANPP in both periods (Fig. 2B). Furthermore, biomass of *L. chinensis* and PB was negatively correlated (Fig. 2C) from 1980–2010.

**Figure 3:**
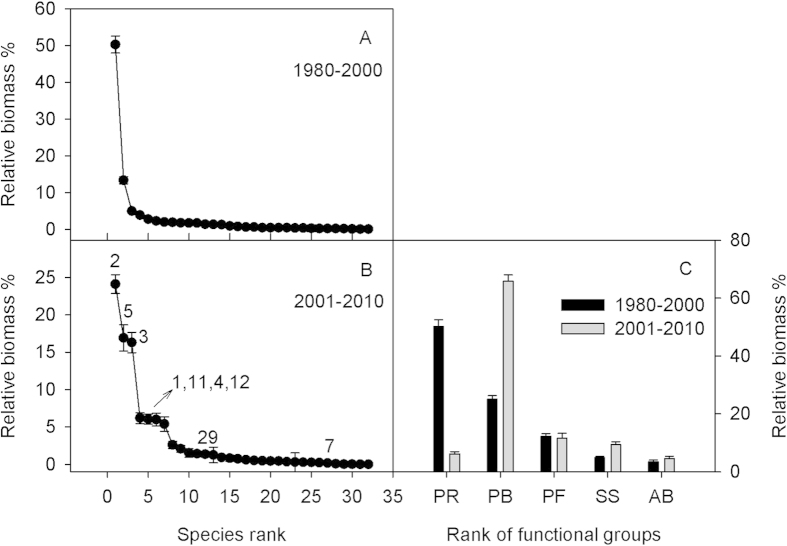
Relative biomass of individual species and functional groups in two periods of 1980–000 and 2001–010. The species were ranked by relative contributions to the total biomass in (**A,B**). The numbers in (**B**) near the dots showed their original rank number in (**A**), which undergoing big change. Species are classified into the following five plant functional groups: perennial rhizomatous grasses (PR, comprised only *Leymus chinensis*), perennial bunchgrasses (PB), perennial forbs (PF), shrubs and semi-shrubs (SS), and annuals and biennials (AB).

The large and rapid reduction in *L. chinensis* biomass also resulted in large change in community structure. During 1980–2000, *L. chinensis* was by far the most dominant member of the community, with *Stipa grandis, Achnatherum sibiricum*, *Carex korshinskyi*, *Agropyron cristatum*, *Caragana microphylia* ranked (by relative biomass) 2–6 in the community (Fig. 3A). These 2–6^th^ ranked species contributed 27% to ANPP, whereas the remaining 26 species in the community contributed 23% to ANPP. However, during 2001–2010, with *S. grandis* becoming the top-ranked species, the increased abundance in *S. grandis*, *A. cristatum* and *A. sibiricum* collectively contributed 57% of ANPP (Fig. 3B). In this second period, *L. chinensis* was relegated to 4^th^ ranked species, and along with *Cleistogenes squarrosa*, *C. korshinskyi*, and *Artemisia frigida* (ranked from 5–7) contributed 24% of ANPP. Several other species also exhibited dramatic changes in relative abundance between the two periods. For example the forb, *Artemisia pubescens* decreased its rank from 7 to 27, while *Chenopodium glaucum* increased its rank from 29 to12. For plant functional groups (see details for species composition of each plant functional group in Bai *et al.* 2004), perennial rhizomatous grasses (PR, comprised of only *L. chinensis*) decreased significantly in abundance (*P* < 0.05), while perennial bunchgrasses (e.g., *S. grandis*, *A. cristatum* and *A. sibiricum*) and shrubs and semi-shrubs (e.g., *Kochia prostrata*) increased significantly (Fig. 3C, *P* < 0.05). The increase of PB compensated for most of the decrease of PR.

**Figure 4:**
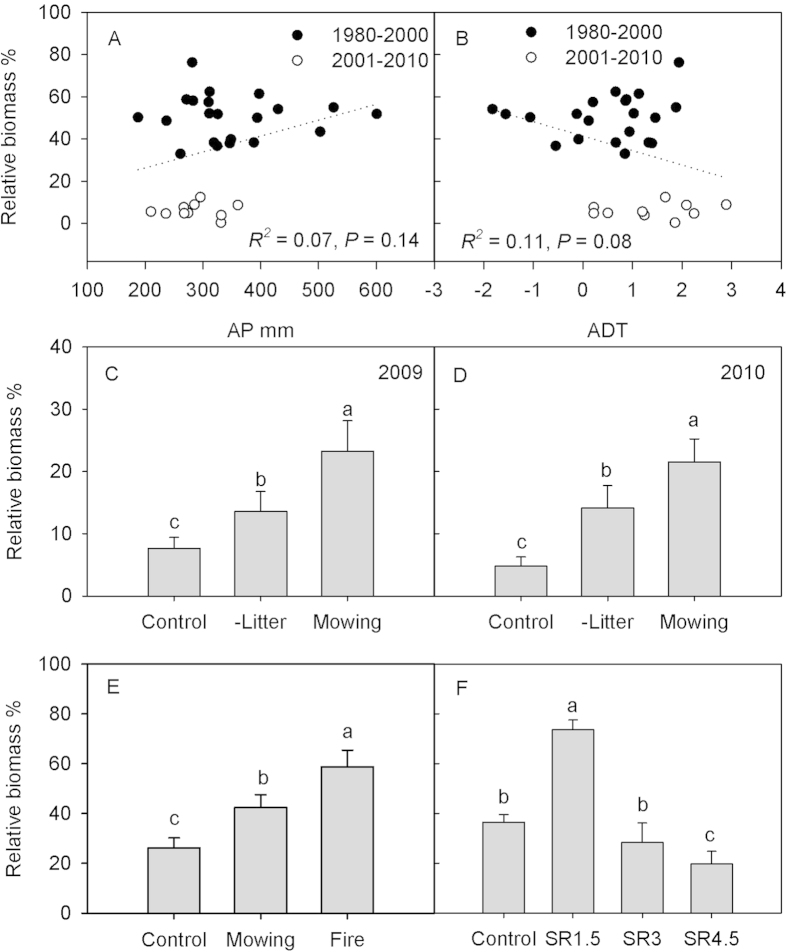
The effects of (A,B) climate (AP = annual precipitation; MDT = mean daily temperature) and (C–F) different disturbances on relative biomass of *L. chinensis* (see text for details). For (**F**), there were four levels of sheep stocking rates (SR) in the grazing experiment: 0, 1.5, 3 and 4.5 sheep ha^−1^. Different letters represent statistically significant differences between treatments at *P* < 0.05 level. Error bars represent SE.

To assess potential mechanisms underlying the dramatic change in abundance of *L. chinensis*, we examined relationships between biomass of *L. chinensis* and annual precipitation or mean daily temperature over the study period. In addition, we utilized several short-term experiments to assess the impacts of litter removal (simulating fire), mowing (simulating grazing) and grazing intensity on *L. chinensis* abundance. Although annual precipitation decreased and mean daily temperature increased significantly from the 1980–2000 period to the 2001–2010 period (*P* < 0.05), dominance of *L. chinensis* was not significantly related with annual precipitation (*P* = 0.14, *r*^2^ = 0.07, Fig. 4A) or mean daily temperature (*P* = 0.08, *r*^2^ = 0.11, Fig. 4B) from 1980–2010 and for both periods. For the experiment removing litter and mowing vegetation at the end of the growing season, dominance (relative biomass) of *L. chinensis* was significantly higher in 2009 and 2010 than undisturbed (control) grassland (*P* < 0.05, Fig. 4C,D). Similarly, relative abundance of *L. chinensis* was enhanced by both early season fire and end-of-season mowing (Fig. 4E), as well as light grazing by sheep (Fig. 4F). However, with high intensity grazing, relative abundance of *L. chinensis* decreased when compared to the ungrazed treatment.

## Discussion

Limiting the frequency of disturbances is a key tool for nature reserve management and ecological restoration^8^,^24^. Yet, we have limited understanding of the long-term (e.g. >30 yrs) consequences of such prevention of disturbance regimes. As expected, the dominant grass species, *L. chinensis*, which can strongly drive ecosystem functions, such as ANPP, declined with fire and grazing exclusion in a semi-arid Inner Mongolian grassland. However, contrary to expectations, this decline was not gradual, but rather considerably lagged and abrupt. The dominance of *L. chinensis* remained relatively stable in the first 21 years of cessation of disturbance in this study, but then *L. chinensis* declined rapidly and was relegated to subdominant status in the community within only a year or two. If our study had lasted less than 21 years, we would have concluded that prevention of disturbance had no significant effects on *L. chinensis*, highlighting the importance of long term studies. Thus, even two decades may not be ‘long-term’ enough for some processes in plant communities.

Although *L. chinensis* dominance declined abruptly, aboveground productivity remained surprisingly unaffected by the dramatic loss of this key grass species. It is well known that for most ecosystems the dominant species mediate most ecological processes^17^,^25^, control the majority of the resources (including space) and have disproportionate impacts on species interactions and ecosystem functions^17^. Consistent with this, we found that the biomass of *L. chinensis* explained 69% variation of ANPP in the first 21 years of the study. However, given their role in communities, changes in abundance of dominant species are expected to impact ecosystem processes and function significantly or even disastrously^17^,^26^,^27^,^28^. But contrary to this expectation, we did not observe large effects on ecosystem function due to compensatory responses of a subset of the community–perennial bunchgrasses (PB, *S. grandis*, *A. cristatum* and *A. sibiricum*)–which were subdominant in the community during the first 21 years of disturbance exclusion. Such compensatory responses have been shown to be important in maintaining ecosystem function in a 24-year study in the same site^29^ and in other ecosystems^20^,^30^,^31^. Our results point to the possibility that compensation may not result from one dominant species being replaced by a functionally similar species (in this case a perennial rhizomatous grass), but that several species may collectively result in compensation and that these species may belong to a completely different functional group – perennial bunchgrasses.

The large shift in abundance of *L. chinensis* may have resulted from a number of factors. Here we explore two potential drivers: gradually changing climate and the long-term exclusion of disturbances. We found annual mean precipitation decreased and annual mean temperature increased significantly from 2001–2010 (*P* < 0.05), consistent with the decrease of *L. chinensis*’ dominance. However, climate and *L. chinensis*’ dominance were not significantly correlated. Furthermore, *L. chinensis* did not decline during the same period of gradually changing climate in an adjacent site which had been fenced since 1999^32^. If climate change induced the decrease of *L. chinensis* in our long-term site from 2001–2010, we would expect to observe the same decrease in adjacent sites. Furthermore, we did not observe any extremes in climate (drought, high heat) during the time of rapid decline of *L. chinensis*. Climate extremes, such as drought, have been shown to be important for triggering abrupt declines in dominant species in other systems^33^. Thus, taken together with the observation from 1980 to 2010, we inferred that neither gradually changing climate nor climate extremes were the primary drivers of the drastic decline of *L. chinensis*.

To assess the role that lack of disturbance played in the change in *L. chinensis* abundance over time, we examined results from several short-term studies manipulating fire and mowing, as well as the presence of litter. Litter removal and mowing increased the dominance of *L. chinensis* in the same site significantly, while in other sites mowing, fire and low-density grazing also increased the dominance of *L. chinensis* significantly. These results clearly show the importance of disturbance in maintaining the dominance of *L. chinensis* in Inner Mongolian semi-arid grasslands. However, another mowing experiment conducted in the same research site showed that the mowing treatment decreased the dominance of *L. chinensis* before 1998^23^, indicating that the same disturbance type could induce different responses if acting in different successional stages. Similarly, intensive grazing decreased the dominance of *L. chinensis*^34^, so the relative degree of disturbance also is an important consideration. The experimental prevention of disturbance underlies the loss of dominance of *L. chinensis* in the 31 year long observation. Thus, it appears that moderate disturbance is critical to maintain ecosystem structure of Inner Mongolian grasslands.

Although these short-term experiments demonstrate that disturbances are important for maintaining dominance by *L. chinensis*, they do not explain the abrupt change in abundance that was observed only after 21 years of exclusion of disturbances. The nonlinear change suggests that a threshold was crossed^22^, but the mechanisms underlying the abrupt change remains unclear. One candidate mechanism is the buildup of litter over the long-term negatively impacted the resprouting of *L. chinensis*, which reproduces primarily asexually via rhizomatous growth. Short-term litter removal increased *L. chinensis* abundance, and therefore it may be that it takes a long time for the buildup of litter to have negative demographic effects on this species. Similar negative effects of litter have been observed for other rhizomatous grasses in the absence of fire and grazing^35^. Another potential mechanism could be that it may take some time for the primarily sexually reproducing bunchgrasses to increase in abundance to a point in which they could negatively impact *L. chinensis*. Finally, changes in resource availability could also be an important mechanism affecting *L. chinensis*, either directly or indirectly by favoring less abundant species^36^. *L. chinensis* is known to have high stoichiometric homeostasis^37^ and low growth rate^38^, which may promote *L. chinensis* under natural disturbance regimes, but accumulation of resources under lack of disturbance could allow other less competitive species to become more abundant over time. Unfortunately, data is not available to fully test these mechanisms, and thus further studies are needed to fully understand the factors that lead to the abrupt shift in *L. chinensis* with exclusion of disturbances over the long-term.

In conclusion, our 31-year study showed that relative biomass of *L. chinensis* declined sharply only after 21 years of exclusion of grazing and fire. Thus, long-term management to prevent disturbances can have large impacts on plant communities but these impacts may be considerably lagged. ANPP did not decline accordingly, suggesting collapse of dominant species does not necessarily result in significant change in ecosystem function, as a consequence of compensatory responses of a functionally different subset of the community – perennial bunchgrasses – which were subdominant in the community, rather than species that were functionally similar. Therefore, it is clear that we need to understand functional roles of less common species in communities, as these often overlooked species may be critical for maintenance of ecosystem function over the long-term with alterations in disturbance regimes and other global changes.

## Material and Methods

We utilized four studies to address our research questions: 1) a long term field monitoring program, 2) a litter removal and mowing experiment, 3) a mowing and fire experiment, and 4) a grazing experiment. All of these studies were located at the Inner Mongolia Grassland Ecosystem Research Station (IMGERS), which is located in the Xilin River Basin, Inner Mongolia Autonomous Region, China (43°38′ N, 116°42′ E). The study area selected for the four studies is dominated by the perennial, rhizomatous C_3_ grass, *L. chinensis*, an important, widely distributed rhizomatous C_3_ grass species that dominates in the eastern part of the Eurasian steppe (40°–62° N, 87°–130° E), a 420,000 km^2^ area. Interspersed in this matrix of grass is diverse suite of uncommon grass and forb species that comprise the following functional groups: perennial bunchgrasses (PB), perennial forbs (PF), shrubs and semi-shrubs (SS), and annuals and biennials (AB). The annual mean temperature in the study area is 0.3 °C with mean monthly temperatures ranging from −21.6 °C in January to 19.0 °C in July. The annual mean precipitation is 346.1 mm with 60–80% falling during the growing season from May to August, and approximately 10% falling as snow.

### Long term field monitoring

The long-term monitoring took place at a 500 × 500 m site, which was fenced to exclude grazers in 1979. Prior to that, the study area was subject to light grazing. The grassland outside the fenced area continued to be grazed with the rate of 1.5–3.0 sheep ha^−1^. There was no fire during our study. As we have no replicate for fenced and grazed area, an east–west transect of 200 * 100 m was established with five equal-sized replicate blocks (40 * 100 m each) within each area to ensure the sampling area is big enough to represent the spatial heterogeneity. From 1980 to 2010, end of season aboveground biomass was sampled by clipping all plants at ground level within a 1 × 1 m quadrat that was randomly located within each block, which decreased the limitation of no replicate for fenced and grazed area. All living vascular plants were sorted to species, oven-dried at 60 °C and weighed. Because the standing crop of these steppe communities reached the annual peak at the end of August, our estimated community biomass approximated the aboveground net primary productivity (ANPP) of these ecosystems. As we sampled ANPP in the end of August for the long-term monitoring study, annual precipitation (AP) and annual mean daily temperature (MDT) collected at the study site were based on data from September 1 of the previous year to August 31 of the current year. All meteorological data were obtained from the weather stations of IMGERS. Full methods and details on experimental treatments can be found in earlier studies^29^,^39^.

### The litter removal and mowing experiment

The litter removal and mowing experiment was initiated in 2007 and consists of the following treatments: control, litter removal, and mowing, each replicated six times (3 treatments × 6 replicates = 18 plots total). The plots were 3 × 3 m, with 1 m buffer separating each plot. The experimental treatments were randomly assigned to each plot. To simulate grazing, a mowing treatment was carried out by clipping all the standing plants at 5 cm above ground on 1 September each year. To simulate the effects of decreased litter after grazing and fire, the litter removal treatment was implemented on 30 August each year by removing all litter at the ground level. Aboveground biomass was sampled within 1 × 1 m quadrats within each plot on 30 August in 2009 and 2010 using the same methods as described above.

### The fire and mowing experiment

The fire and mowing experiment was established in 2005 and included three treatments: control, fire and mowing. The experimental design was a randomized block design, with 6 blocks (each separated by a 2-m walkway), with each block containing three 10 × 10 m plots, each separated by a 1-m buffer. The treatments were randomly assigned to the three plots within each block. All blocks were located at the same topographic position and on similar soils. The fire treatments were applied in early May of 2006 and 2007. The fire removed almost all the aboveground plant materials and surface litter. The mowing treatment was implemented in October 2006, with all the plants cut at 5 cm above ground. On 15 August 2007, aboveground biomass was sampled by cutting all plants at ground level within a 1 × 0.5 m quadrat located within each plot. All living vascular plants were sorted to species, oven-dried at 60 °C and weighed.

### The grazing experiment

In 2004, we established a grazing experiment consisting of seven stocking rates^40^. For this study, we utilized only four of the stocking rate treatments: 0, 1.5, 3.0 and 4.5 sheep ha^−1^ (hereafter referred to as control, SR1.5, SR3.0 and SR4.5, respectively). The area of each fenced plot was 2 ha except for treatment SR1.5, in which was 4 ha. Starting in 2005, sheep were transferred to plots in mid-June and were maintained there until mid-September of each year. We sampled the plants within a 1 × 1 m quadrat with 6 replicates in each plot in August 2007.

### Statistical analysis

Linear regression analysis was used to assess the relationships between biomass of *L. chinensis*, biomass of PB and ANPP, as well as relationships between relative biomass (calculated as the ratio of biomass of *L. chinensis* to ANPP) and biomass of *L. chinensis* and AP and MDT. ANOVA was used to test the difference of annual precipitation, mean daily temperature, relative biomass of functional groups, and relative biomass of *L. chinensis* among various treatments for each experiment separately. All analyses were performed using SAS (version 9.0, SAS Inst., Cary, NC, USA).

## Additional Information

**How to cite this article**: Yu, Q. *et al.* Long term prevention of disturbance induces the collapse of a dominant species without altering ecosystem function. *Sci. Rep.* **5**, 14320; doi: 10.1038/srep14320 (2015).

## References

[b1] Sala O. E. *et al.* Biodiversity—Global biodiversity scenarios for the year 2100. Science 287, 1770–1774 (2000). 10710299 10.1126/science.287.5459.1770

[b2] Vitousek P. M., Mooney H. A., Lubchenco J. & Melillo J. M. Human Domination of earth’s ecosystems. Science 277, 494–499 (1997).

[b3] Pickett S. T. A., Kolasa J., Armesto J. J. & Collins S. L. The ecological concept of disturbance and its expression at various hierarchical levels. Oikos 54, 129–136 (1989).

[b4] Collins S. L. Disturbance frequency and community stability in native tallgrass prairie. Am Nat 155, 311–325 (2000). 10718728 10.1086/303326

[b5] Cramer W. *et al.* Global response of terrestrial ecosystem structure and function to CO_2_ and climate change: results from six dynamic global vegetation models. Global Change Biol 7, 357–373 (2001).

[b6] Schroter D. *et al.* Ecosystem service supply and vulnerability to global change in Europe. Science 310, 1333–1337 (2005). 16254151 10.1126/science.1115233

[b7] Keitt T. H. Coherent ecological dynamics induced by large-scale disturbance. Nature 454, 331–334 (2008). 18633416 10.1038/nature06935

[b8] Benayas J. M. R., Newton A. C., Diaz A. & Bullock J. M. Enhancement of biodiversity and ecosystem services by ecological restoration: A Meta-analysis. Science 325, 1121–1124 (2009). 19644076 10.1126/science.1172460

[b9] Palmer M. A. & Filoso S. Restoration of ecosystem services for environmental markets. Science 325, 575–576 (2009). 19644112 10.1126/science.1172976

[b10] Burns C. E., Collins S. L. & Smith M. D. Plant community response to loss of large herbivores: comparing consequences in a South African and a North American grassland. Biodivers Conserv 18, 2327–2342 (2009).

[b11] Veen G. F., Blair J. M., Smith M. D. & Collins S. L. Influence of grazing and fire frequency on small-scale plant community structure and resource variability in native tallgrass prairie. Oikos 117, 859–866 (2008).

[b12] Verdu J. R., Crespo M. B. & Galante E. Conservation strategy of a nature reserve in Mediterranean ecosystems: the effects of protection from grazing on biodiversity. Biodivers Conserv 9, 1707–1721 (2000).

[b13] Hobbs R. J., Yates S. & Mooney H. A. Long-term data reveal complex dynamics in grassland in relation to climate and disturbance. Ecol. Monogr. 77, 545–568, (2007).

[b14] Lamb E. G. Direct and indirect control of grassland community structure by litter, resources, and biomass. Ecology 89, 216–225 (2008). 18376563 10.1890/07-0393.1

[b15] Pickett S. T. A. & White P. S. The ecology of natural disturbance and patch dynamics. (Academic Press, 1985).

[b16] Bormann D. B. & Likens G. E. Pattern and process in a forested ecosystem. (Springer-Verlag, 1979).

[b17] Grime J. P. Benefits of plant diversity to ecosystems: immediate, filter and founder effects. J Ecol 86, 902–910 (1998).

[b18] Smith M. D. & Knapp A. K. Dominant species maintain ecosystem function with non-random species loss. Ecol Lett 6, 509–517 (2003).

[b19] Hillebrand H., Bennett D. M. & Cadotte M. W. Consequences of dominance: A review of evenness effects on local and regional ecosystem processes. Ecology 89, 1510–1520 (2008). 18589516 10.1890/07-1053.1

[b20] Allan E., Weisser W., Weigelt A., Roscher C., Fischer M. & Hillebrand H. More diverse plant communities have higher functioning over time due to turnover in complementary dominant species. P Natl Acad Sci USA 108, 17034–17039 (2011). 10.1073/pnas.1104015108 PMC3193239 21949392

[b21] Sasaki T. & Lauenroth W. K. Dominant species, rather than diversity, regulates temporal stability of plant communities. Oecologia 166, 761–768 (2011). 21279386 10.1007/s00442-011-1916-1

[b22] Smith M. D., Knapp A. K. & Collins S. L. A framework for assessing ecosystem dynamics in response to chronic resource alterations induced by global change. Ecology 90, 3279–3289 (2009). 20120798 10.1890/08-1815.1

[b23] Bao Y. J., Li Z. H. & Zhong Y. K. Compositional dynamics of plant functional groups and their effects on stability of community ANPP during 17 yr of mowing succession on Leymus chinensis steppe of Inner Mongolia, China. Acta Bot Sin 46, 1155–1162 (2004).

[b24] Margules C. R. & Pressey R. L. Systematic conservation planning. Nature 405, 243–253 (2000). 10821285 10.1038/35012251

[b25] Whittaker R. H. Dominance and diversity in land plant communities: numerical relations of species express the importance of competition in community function and evolution. Science 147, 250–260 (1965). 17788203 10.1126/science.147.3655.250

[b26] Sala O. E., Lauenroth W. K., McNaughton S. J., Rusch G. & Zhang X. [Temperate grasslands] Global Biodiversity Assessment [ Mooney H. A., Lubchenco J., Dirzo R. & Sala O. E. (ed.)] [361–366] (Cambridge University Press, 1995).

[b27] Scheffer M., Carpenter S., Foley J. A., Folke C. & Walker B. Catastrophic shifts in ecosystems. Nature 413, 591–596 (2001). 11595939 10.1038/35098000

[b28] Briske D. D., Fuhlendorf S. D. & Smeins F. E. A unified framework for assessment and application of ecological thresholds. Rangeland Ecol Manag 59, 225–236 (2006).

[b29] Bai Y. F., Han X. G., Wu J. G., Chen Z. Z. & Li L. H. Ecosystem stability and compensatory effects in the Inner Mongolia grassland. Nature 431, 181–184 (2004). 15356630 10.1038/nature02850

[b30] Gonzalez A. & Loreau M. The causes and consequences of compensatory dynamics in ecological communities. Annu Rev Ecol Evol S 40, 393–414 (2008).

[b31] Roscher C. *et al.* Identifying population- and community-level mechanisms of diversity-stability relationships in experimental grasslands. J Ecol 99, 1460–1469 (2011).

[b32] He N., Han X., Yu G. & Chen Q. Divergent changes in plant community composition under 3-decade grazing exclusion in continental steppe. Plos One 6, e26506 (2011). 22073169 10.1371/journal.pone.0026506 PMC3206806

[b33] Allen C. D. & Breshears D. D. Drought-induced shift of a forest–woodland ecotone: Rapid landscape response to climate variation. P Natl Acad Sci USA 95, 14839–14842 (1998). 10.1073/pnas.95.25.14839 PMC24536 9843976

[b34] Wang R. Z. & Ripley E. A. Effects of grazing on a *Leymus chinensis* grassland on the Songnen plain of north-eastern China. J Arid Environ 36, 307–318 (1997).

[b35] Knapp A. K. & Seastedt T. R. Detritus accumulation limits productivity of tallgrass prairie. Bioscience 36, 662–668 (1986).

[b36] Knapp A. K., Briggs J. M., Hartnett D. C. & Collins S. L. Grassland dynamics: long-term ecological research in tallgrass prairie. (Oxford University Press, 1998).

[b37] Yu Q. *et al.* Stoichiometric homeostasis of vascular plants in the Inner Mongolia grassland. Oecologia 166, 1–10 (2011). 21221646 10.1007/s00442-010-1902-z

[b38] Yu Q. *et al.* Testing the growth rate hypothesis in vascular plants with above- and below-ground biomass. Plos One 7, e32162 (2012). 22427823 10.1371/journal.pone.0032162 PMC3302800

[b39] Yu Q. *et al.* Linking stoichiometric homoeostasis with ecosystem structure, functioning and stability. Ecol Lett 13, 1390–1399 (2010). 20849443 10.1111/j.1461-0248.2010.01532.x

[b40] Wu H. *et al.* Feedback of grazing on gross rates of N mineralization and inorganic N partitioning in steppe soils of Inner Mongolia. Plant Soil 340, 127–139 (2011).

